# Assessment of the Accuracy of a Multi-Beam LED Scanner Sensor for Measuring Olive Canopies

**DOI:** 10.3390/s18124406

**Published:** 2018-12-13

**Authors:** Rafael R. Sola-Guirado, Sergio Bayano-Tejero, Antonio Rodríguez-Lizana, Jesús A. Gil-Ribes, Antonio Miranda-Fuentes

**Affiliations:** 1 Rural Engineering Department, University of Cordoba. Ed. Leonardo da Vinci, Campus Rabanales, Ctra. Nacional IV, km 396, 14014 Córdoba, Spain; ir2sogur@uco.es (R.R.S.-G.); p52bates@uco.es (S.B.-T.); gilribes@uco.es (J.A.G.-R.); 2 Aerospace Engineering and Fluid Mechanics Department, University of Seville, Ctra. de Utrera km 1, 41013 Sevilla, Spain; arodriguez2@us.es

**Keywords:** laser scanner, accuracy test, high-growing crops, canopy characterization, olive orchards, agricultural work

## Abstract

Canopy characterization has become important when trying to optimize any kind of agricultural operation in high-growing crops, such as olive. Many sensors and techniques have reported satisfactory results in these approaches and in this work a 2D laser scanner was explored for measuring canopy trees in real-time conditions. The sensor was tested in both laboratory and field conditions to check its accuracy, its cone width, and its ability to characterize olive canopies in situ. The sensor was mounted on a mast and tested in laboratory conditions to check: (i) its accuracy at different measurement distances; (ii) its measurement cone width with different reflectivity targets; and (iii) the influence of the target’s density on its accuracy. The field tests involved both isolated and hedgerow orchards, in which the measurements were taken manually and with the sensor. The canopy volume was estimated with a methodology consisting of revolving or extruding the canopy contour. The sensor showed high accuracy in the laboratory test, except for the measurements performed at 1.0 m distance, with 60 mm error (6%). Otherwise, error remained below 20 mm (1% relative error). The cone width depended on the target reflectivity. The accuracy decreased with the target density.

## 1. Introduction

Tree crops are attracting the attention of many researchers because of the difficulty of their management [1,2,3,4,5,6,7,8]. In contrast to the arable crops, whose mechanization and management is very controlled, tree crops present the difficulty of being tridimensional, which crucially conditions the way many operations are performed. This circumstance differs according to tree morphology, from existing hedgerow orchards that behave geometrically similar to regular leaf walls, such as the case of apple trees and vineyards of central Europe [9,10], to traditional orchards with big-sized irregular isolated trees, such as the citrus and olive plantations of Southern Europe [11,12]. Therefore, knowing canopy characteristics is very important from a management point of view and, thus, canopy characterization is of remarkable importance in most operations performed in these crops, such as harvesting, pruning, spraying, and every related operation performed on the tree crown.

Canopy characterization is attracting increasing attention because of the need to obtain specific knowledge about specific parameters related to tree size and geometry, so as to adapt machines to operate automatically [13,14,15,16,17,18,19,20]. Each particular case requires different information, involving a diverse degree of resolution and measurement range, so there is no unique characterization methodology that matches every single situation [21]. These methodologies significantly differ depending on the tree plantation pattern. Thus, in hedgerow/trellis orchards, the basic measurements taken are oriented towards the quantification of the total tree volume per hectare or the tree height. In the case of isolated trees, on the other hand, efforts are conducted to obtain the individual tree crown volume.

The different methodologies in use for canopy characterization can be classified as manual and electronic [12,22]. The manual methodologies are diverse, but in every case, they involve operators taking measurements with different kinds of equipment, such as topographic milestones and measuring tapes. The base of these methods relies on an operator standing next to the tree with the measurement instrument and another one standing beside the first operator and noting down the measurements they take. In other occasions, destructive operations are required, for example for taking leaf samples to characterize the leaf density [7,9,23]. These methodologies present the advantage of being easy to use, allowing farmers and technicians to implement them in commercial plantations without an important degree of training but, on the other hand, they are not extremely precise and, in addition, they sometimes require a lot of work, especially if many trees require characterization.

Among the different manual methods used by authors with scientific purposes, the ellipsoid method is the most frequent [12,24,25]. Other important methods are the manual measurement of hedgerow trees, usually involving the measurement of the wall height and width, [26] the crown vertical projection method, [27] consisting of determining the ground projection of the tree crown in isolated trees, subsequently estimating the crown volume through previously developed correlations, the tree silhouette method [12], based on manually defining the tree crown contour on different pictures taken from the tree, determining its area and determining the tree volume by revolutionizing this area around the central symmetry axle, and the mean vector method, recently proposed for manual olive crown characterization by Miranda-Fuentes et al. [12].

On the other hand, electronic methods involve the use of different sensors to obtain, depending on the case, several parameters of the canopy geometry. The information that the sensors provide may be from drones [28], from satellite imagery [29], or from the ground [30]. The main advantages of these systems are their precision and their automatic work but, on the other hand, they usually need specific training in onboard electronics, image analysis or programming to access all their potential). They are also expensive in general, so their use is limited to research and big farms for the moment, though their price is continuously decreasing, making them more affordable every day.

Generally, most of the methods included in this group involve distance measurement, though some are based on photography and image analysis for direct volume calculation, such as the case of stereo vision methods [31,32]. These distances allow for the establishment of point sets in three-dimensional environments, which enable the determination of canopy profiles, [33], height, [9] volume [25,34,35], or even density [36,37,38]. The most frequent electronic methodologies involve the use of ultrasonic sensors, [39,40,41] light sensors, [42] radar systems [43] and LiDAR (Light Detection and Ranging) sensors [44,45]. Among all of the aforementioned methods, the LiDAR scanners have been proved to have the best accuracy [8]. The advantage of these systems is that they sweep a nearly continuous angular range (angular resolution usually lower than 1°), emitting lasers in a range of directions (usually 360°) in their working plane, so they can scan any surface, obtaining its contour. For the volume determination, a third dimension is needed (an additional movement perpendicular to the mentioned working plane). Here we have two additional options or categories to classify LiDAR systems: 3D and 2D LiDAR. In the case of the 3D LiDAR, this movement is achieved with an additional rotation of the whole scanner instrument around its vertical axle, not only sweeping a vertical plane but a horizontal one as well [46]. The 2D LiDAR, on the other hand, lacks this second horizontal rotation, so if left static, it only characterizes a 2D point set. In this case, the movement needs to be added, and in agricultural applications the sensor is attached to a vehicle that moves alongside the tree row, scanning it from its both sides [47].

In this way, different authors have successfully characterized very different crops, such as apple [39], peach [16], citrus [36], olive [12,46] or vineyard [48]. The main problems regarding LiDAR use in field conditions is the need for advanced training in data management and electronics. Maybe in the future these sensors can be prepared to operate automatically but, for the moment, they are limited to research and very specific applications. Similar problems apply to short-distance photogrammetry techniques, that, although they would be another interesting method for measuring distances based on·3D models, would require a post-analysis phase, and so they are not feasible for real-time application.

In the particular field of the pesticide application, where the canopy characterization presents a particular interest to adapting pesticide doses to requirements in terms of leaf surface to be covered [49] and accomplishing, in this way, the sustainable use of agrochemicals demanded by Directive 2009/128/CE [50], the only sensors commercially implemented are ultrasonic sensors. Considering olive, the most important tree crop in the European Union with a total harvested area of around 5 Mha and an annual production of nearly 12 Mt, the use of these sensors is only limited to an ON/OFF function, i.e., they are intended to be used as a switch for the electrovalves controlling the spray section of the airblast sprayer [16,51]. This function has been shown to provide great savings when operating in other crops [52,53] and even in this one [54].

The aim of the presented research is to explore a multi-beam LED (Light-Emitting Diode) scanner with a real-time approach, but without compared to other types of sensors of techniques. The main objectives were to determine the accuracy of the sensor according to different parameters, i.e., the reflectivity of the material and the gaps percentage, to measure the width of each sensor’s measuring cone at different distances and to test the sensor’s accuracy under real field conditions in isolated and hedgerow olive orchards.

## 2. Materials and Methods

### 2.1. Measuring System

**Figure 1:**
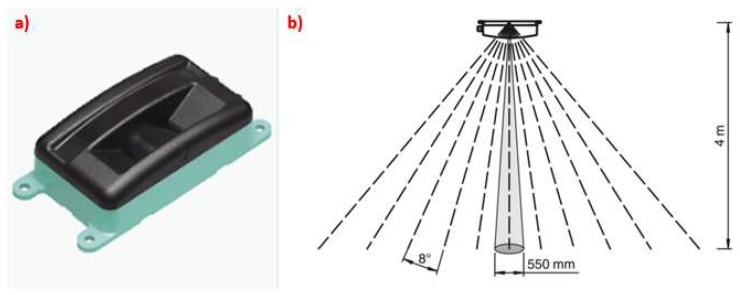
(**a**) Laser scanner OMD8000-R2100-B16-2V15. (**b**) Arrangement of the measurement spots.

The sensor used to characterize the canopy was the 2D laser scanner OMD8000-R2100-B16-2V15 (Pepperl + Fuchs, Mannheim, Germany; Figure 1a). This device counted using a CAN open Interface and measuring method PRT (Pulse Ranging Technology). The sensor consists of a transmitter and a receiver placed in the same housing. The transmitter emits pulses of infrared light that are reflected on the target and captured back by the receiver. The distance is calculated through the time takes the pulse emitted to return to the receiver and the speed of light (c).

The sensor counted using 11 emitter elements arranged side by side, which can span a scanning range of 88 degrees. The main difference to a conventional laser scanner is the capability to take the measurement of a spot rather than a single point, in a similar way to the ultrasonic sensor, commonly used for canopy characterization. According to the manufacturer, the diameter of the light spot is 550 mm at 4 m distance (Figure 1b). The measuring range of the sensor was 0.2 to 8.0 m (relative humidity supported up to 90%) with IR (Infrared Radiation) light source in modulated infrared of 850 nm.

**Figure 2:**
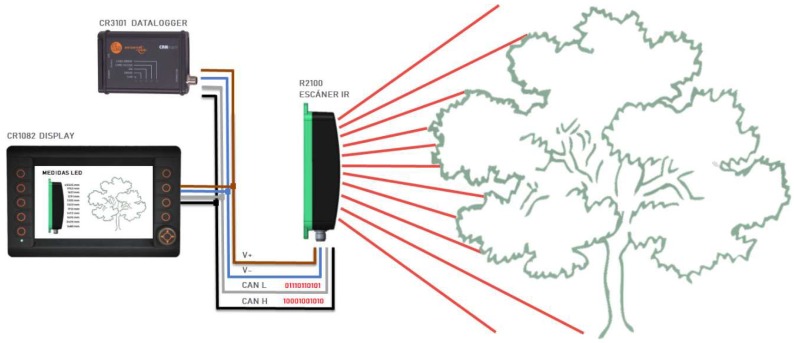
Electronic arrangement of the 2D LiDAR scanner.

For the laboratory and field measurements, the sensor was mounted on a vertical tripod that enabled the regulation of the horizontal and vertical angles (though two bubble levels) and the sensor height. It also incorporated different angle indicators to make possible the perfect alignment of the sensor with respect to the horizontal plane. Visual references were also included to make it possible to predict, according to the sensor orientation, every emitter’s direction. The full electronic arrangement is shown in Figure 2.

The sensor was connected to a display (CR1082, IFM Electronic, Essen, Germany) for real-time monitoring of the data from the 11 emitters to detect possible errors or problems. There was a datalogger (CR3101, IFM Electronic, Essen, Germany) that stored the data acquired by the sensors in a “.csv” format file. The scan frequency was 50 Hz, while the acquisition and registry were made at 2 Hz since it was the maximum allowed by the datalogger. The measured value noise was 20 mm (1 sigma, 4 m on white, orthogonal) with an angle resolution of 8° and absolute accuracy of ±50 mm (orthogonal).

The data acquisition was pre-programmed in CoDeSys (Controller Development System) language and controlled through the display. After the tests, the data was exported to a laptop computer (Thinkpad Z510, Lenovo, Pekin, China), where it was imported in Microsoft Excel^®^ (Microsoft, Redmond, WA, USA) for preliminary analysis and data visualization. The CoDeSys library was employed to register the data by creating a “.csv” file composed by 8 columns in byte format. Thus, each LED measurement was separated into 2 bytes by programming datalogger and, consequently, each “.csv” file could contain four LED measurements over time. Thus, 3 files were necessary to register 11 LED measurements. Once the data was stored in the computer, the “.csv” files were linked, and all the measurements were converted to original format for analysis using Microsoft Excel^®^. Every single file was created for a given test, an R-software script developed with GPL License RStudio version r243 was used to compile every single one into a general table, containing the different variables organized in different columns, ready to their exportation to the statistical software. There was a code to properly identify data coming from the different trials previously described.

Data was also pre-visualized and collected through the acquisition program developed in CoDeSys language. The data acquisition frequency of 50 Hz allowed for a near real-time monitoring of the 11 measurements. 

### 2.2. Laboratory Arrangement

The laboratory tests were carried out using a large white wall. These tests were aimed at studying the sensor’s accuracy under certain circumstance which could affect its performance in field operations, such as the varying target density. In every test, the measurement distance was a variable. Its maximum value was set at 5 m in every case, as this is the maximum distance usually found in canopy characterization operations in olive orchards when placing the sensor in the center of the space between tree rows. The laboratory arrangement for every test is described in this section.

#### 2.2.1. Absolute and Relative Accuracy of the Sensor

**Figure 3:**
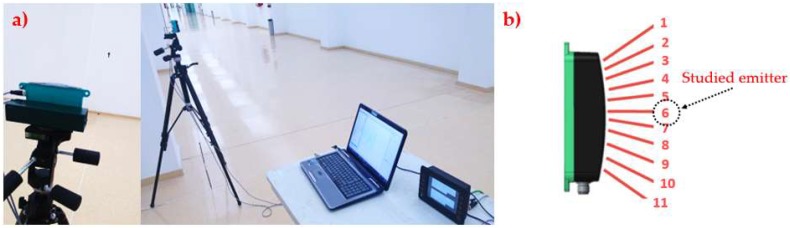
(**a**) Sensor arrangement for accuracy laboratory test, (**b**) tested LED emitters.

The absolute and relative accuracy of the sensor was checked by setting the sensor (on the tripod) horizontal at 1.40 m height, perpendicularly aiming at a horizontal matte white flat wall (Figure 3a). The sensor was placed at a varying distance from the wall, ranging from 0.5 to 5.0 m, in intervals of 0.5 m. To keep the same measurement position in the target, a laser indicator from the laser telemeter (GLM 50 Professional, BOSCH, Chicago, IL, USA) was used. This telemeter, with measurement range from 50 to 50,000 mm and precision of ±1.5 mm, was also used to measure the real distance to the wall, to make the comparison with the measurements taken by the sensor. This sensor has been successfully used in previous studies with similar purposes [22].

To have a reference for the tripod position, marks were set on the ground to follow a straight line when approaching the sensor to the wall. These marks included the sensor’s position in every studied distance, and a weight mass under the tripod allowed for the accurate placement of the tripod to match the sensor’s position.

In laboratory test, and in the field test, acquisition of 11 emitters was taken over 500 ms intervals during a 10 s period. However, by comparison, the laboratory test was carried out by using only the central emitter (emitter 6, Figure 3b) because of spacing restriction.

#### 2.2.2. Cone Width Measurement

Each emitter does not behave like a laser beam in the sense of measuring a punctual distance, but makes a sort of “mean distance measurement” of an area supposed to be circular or ellipsoidal, which is called a “spot”. As can be observed in Figure 1b, this spot increases in diameter when increasing the distance from the sensor, so the LED produces a conical measurement shape that is known as a “measurement cone”. A similar phenomenon can be found in the aforementioned ultrasonic sensors, Ref. [22,39] producing significant interference between measurements of sensors placed very close. Nevertheless, the cone width has a major influence in this interference, as the wider the cone, the higher the area detected in the measurement. It also has major influence on the sensor’s accuracy when measuring the distance to a target point, as the bigger the spot, the lower the certainty of measuring any particular distance. For these reasons, the cone width of one LED emitter was measured. It was also necessary to check if the cone width was symmetric in its two axis or, on the contrary, there was any asymmetry.

**Figure 4:**
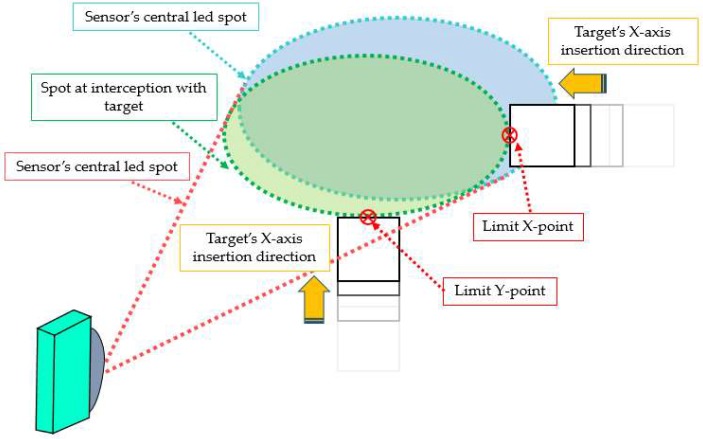
Scheme of the characterization of the cone width.

To measure the cone width in both axes (*x* and *y* in Figure 4), two 500 × 500 mm targets were used. They differed in the material, with the main difference of being reflective or not. This proved to have a significant effect on the cone shape in ultrasonic sensors in previous studies [22]. As with the aforementioned case, only the central emitter was tested, (number “6” in Figure 3b), as every sensor was supposed to behave in the same way. The cone shape was measured in different distances to the sensor. Thus, measurements were taken at 0.5, 1.0, 1.5, 2.0, 2.5, 3.0, 3.5, 4.0, 4.5 and 5.0 m distances. The cone width in each axis was measured as explained in Figure 4. The target was inserted in the emitter’s field of view from the four outermost positions defining the spot size: both sides and the upper and lower part.

With the purpose of ensuring the accuracy of the measurements, the target was mounted on a fixed platform that moved along a straight trajectory in a continuous way. The cone width was determined by stopping the target’s advance in that moment in which the measurement collected by the sensor for the selected emitter changed from the one corresponding to the wall to that to the target, with a difference in between them of 23 cm (Figure 4). This change in the measurement was quickly detected on the computer, by using the data acquisition program, which enabled real-time data visualization, and was noted. Repeating the process 3 times yielded, consequently, 12 points per sampling distance.

#### 2.2.3. Density Influence on Sensor’s Accuracy

One of the most important requirements for a sensor working on detecting a real canopy is its capability to detect the outermost part of the tree crown without suffering excessive accuracy loss because of the low leaf density (i.e., the laser bean penetrating inside the canopy and producing mistakes in the measurement of the canopy contour). In the particular case of the evaluated sensor, it has the capability to measure spots with every one of its emitters. Nevertheless, and as happens in the case of the ultrasonic sensors, [22] it is necessary for the sensor to achieve a minimum target area to have reflected enough energy to detect the object, passing through it in the opposite case. Therefore, it was necessary to know the minimum surface density to reflect the sensor’s signal to predict its possibility to work in different olive plantation systems, with varying leaf density. Thus, the measurement accuracy of the sensor to a density-variable target was evaluated.

**Figure 5:**
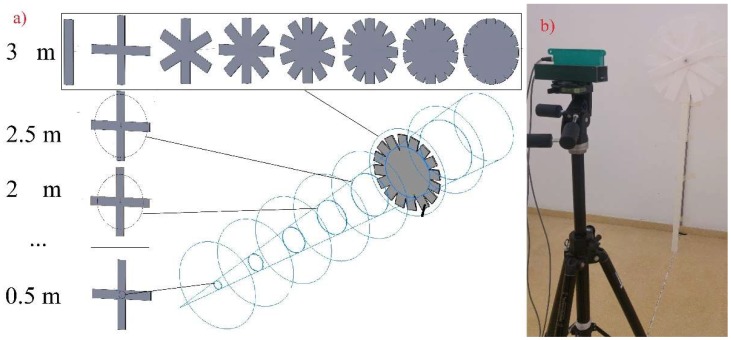
(**a**) Variable-density target for density’s influence on accuracy laboratory test, with the theoretical cone proposed by the manufacturer. (**b**) Laboratory layout.

The target was made of 8 1000 × 150 mm pieces of plastic material (polyethylene) that were combined to achieve high or low density when using more or fewer pieces and fixed to a vertical mast (Figure 5b). Therefore, as it can be seen in Figure 5a, the target ranged from 1 (lowest density) to 8 (highest density) plastic pieces, and the studied distances from the sensor to the target ranged from 0.5 to 5.0 m, with a 0.5 m increment between two consecutive distances. The target was perfectly aligned with sensor number “6” (Figure 3b), by using a laser indicator that helped to correct any possible misalignment in the studied LED (Figure 5b).

To properly correlate the sensor’s accuracy to the target density, the area covered by the target was compared to the total target area with the eight plastic pieces, calculating the independent variable “Target Density” (TD), whose maximum value was 100% (8 pieces) and its minimum was 16.8% (1 piece). The sensor’s absolute and relative error was correlated with this TD to determine the influence of this parameter on the measurement accuracy. For every configuration, a total of 20 distance measurements were taken, and when every combination was completed, the process began consecutively, so that 3 replications of the trial were done in total, which made 60 measurements per single configuration.

### 2.3. Field Arrangement

The field trial was carried out in the experimental olive farm of the University of Cordoba (37°56′00″ N; 4°43′09″ W), which contains two different cultivation patterns, both used in this trial. On the one hand, there was an intensive cultivation pattern, characterized by isolated small-sized trees with row spacing of 7 m and tree spacing 6 m and different olive varieties. On the other hand, there is a super-intensive pattern, with a hedgerow-type orchard, with row spacing of 5 m and tree spacing of 2 m and composed of different varieties.

**Figure 6:**
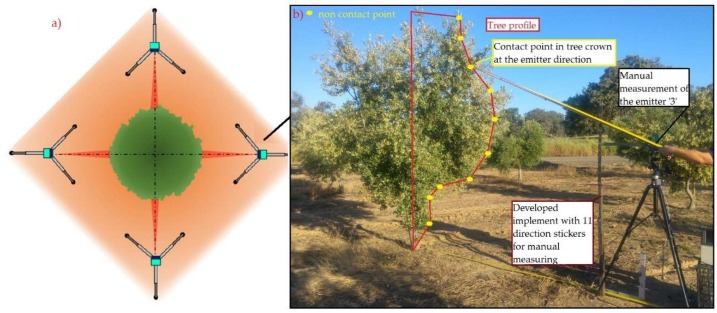
(**a**) Measurement location in the isolated tree test. (**b**) Use of the implement for measuring the contact point in tree at the emitter direction.

**Figure 7:**
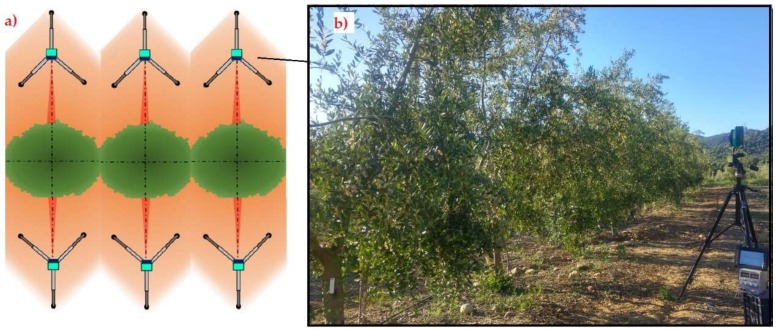
(**a**) Measurement location in the hedgerow trees. (**b**) Sensor measurements.

Trees were measured for isolated trees (Figure 6) and for hedgerow trees (Figure 7) by using the sensor along with its tripod set at 2.5 m distance from the target tree crown. This distance was selected to match the geometry of the sensor working on an intensive cultivation system, in which the row spacing usually ranges from 6 to 8 m, and the total tree height nearly reaches 4 m, simulating the placement of the sensor in a tractor-driven machine, for example an airblast sprayer. The sensor’s height was fixed at 150 cm to coincide with the medium height of the tree crown (manually measured), and properly stabilized. The sensor’s aforementioned height, therefore, was set in relation to the base height of the tree’s trunk in every case to avoid the influence of the tree ridge, which varied in size from one tree to another.

For isolated trees, a total of 6 trees were completely characterized, with 4 sampling positions (profiles) each. For hedgerow trees, 20 trees belonging to two different rows were characterized with 2 sampling positions from each tree side. The measurement point in every tree was that coincident with the medium plane, for generally being the maximum-width profile. 30 measurements were taken for every sampling position and with every single emitter.

To compare each sensor’s measurement with the real distance, an implement was developed to support a rigid stick that enabled the real distance measurement on each emitter (Figure 6). This implement consisted of a fixed metallic platform that contained one support called direction sticker, which held the stick in a second point, being the first point the sensor placement in every case. The two mentioned points defined a line, coincident with the orientation of the longitudinal axle of the measurement cone for the studied emitter. Thus, the rigid stick could have as many orientations as the directions defined by the sensors contained in the scanner (11 in total). The separation between consecutive directions was, therefore, 8°, the angular separation between consecutive emitters (Figure 1b). At the same time, measurements were manually taken with a rule to determine the width of the tree and its canopy height.

The first purpose was to determine the accuracy of the sensor when aiming at real olive trees in field conditions. Therefore, the same electronic layout used in the laboratory was kept (Figure 2). The accuracy of the sensor was checked as the absolute error, calculated as the difference between the real and the sensor’s measurements, expressed in cm. The relative error was also checked, as expressed in Equation (1).

(1) $$RE=\overset{n}{\underset{i=1}{\sum }}\left(\frac{\left|d_{real-i}-d_{sensor-i}\right|}{d_{sensor}-i}\times 100\right)\times \frac{1}{n}$$

where *n* is the number of emitters, 11 in this case, *d_real−i_* is the real distance measured with the rigid stick for the position corresponding to the emitter “*i*“ (cm) and *d_sensor−i_* the distance measured by this emitter “*i*” (cm).

The individual error of an individual emitter “*i*” was calculated, therefore, as explained in Equation (2).

(2) $$RE_{i}=\frac{\left|d_{real-i}-d_{sensor-i}\right|}{d_{sensor}-i}\times 100$$

The measurements with values of 65,535 denoted that pulses of infrared light were not received back to the sensor. Numbers of data value 65,535 were analyzed versus the number of different data value for 20 measurements taken for each LED. If number of 65,535 data value was higher, the LED was directed at a gap or sky. If the number of data values different to 65,535 is higher, the data in the minority were erased from the dataset and not analyzed. Wrong data were identified through filters using Microsoft Excel^®^.

## 3. Results

### 3.1. Laboratory Results

#### 3.1.1. Sensor’s Accuracy Assessment

**Figure 8:**
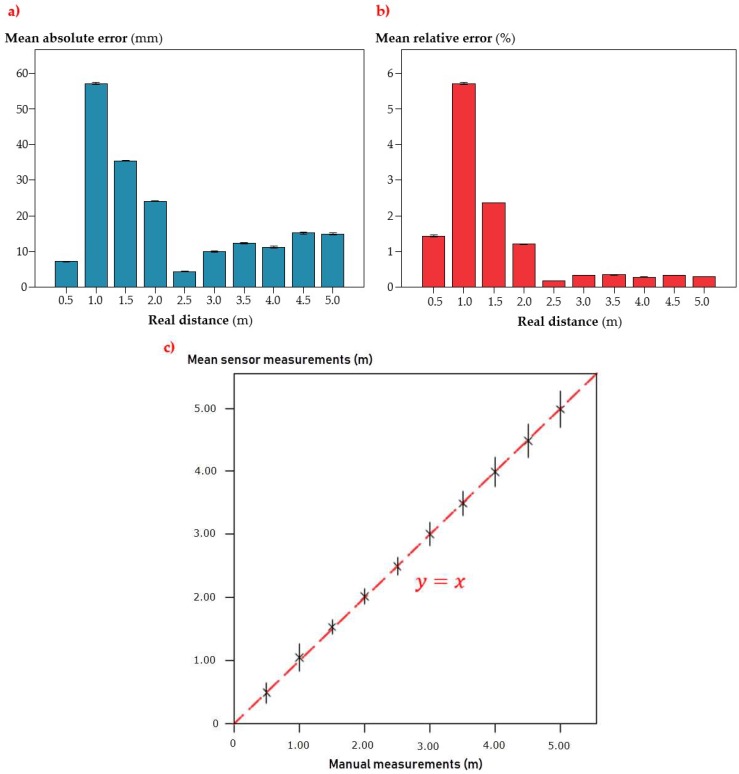
Sensor measurement (**a**) absolute and (**b**) relative mean error. Error bars represent the Standard Error (**c**) Comparison between manual and sensor-collected measurements in the accuracy test. Error bars represent 1000 (10^3^) fold Standard Error.

The sensor’s absolute and relative error for different sampling distances and for emitter 6 is represented in Figure 8a,b.

The sensor was very accurate for its purpose, with low absolute error rates, which were below 60 mm in every case (Figure 8a). This error showed a peak at 1.0 m distance (±58 mm) and then got reduced to a nearly constant value below 15 mm from 2.5 m distance. The minimum absolute error was found at 2.5 m distance, with a mean value of ±5 mm. The irregular pattern described by the results is noticeable: a rising error rate which increases to its maximum value, then decreases, and finally turns stable. This scenario is, of course, well represented in the relative error behavior (Figure 8b). The low relative error rate generated by the sensor, which stayed below 6% for every sampling distance, was expected from the low absolute error values previously mentioned. This performance can be explained by the fact that the target was completely homogeneous and plane, with controlled conditions of light and without environmental dust, which can significantly improve the accuracy of measurements in comparison with a real field environment.

**Figure 9:**
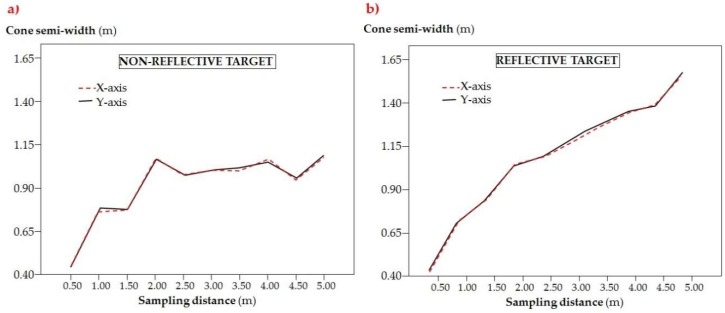
Cone semi width (m) for non-reflective target (**a**) and reflective target (**b**).

When looking at the graph, there is a critical point in which the sensor showed the poorest relative performance: 1 m distance. This was expected from the absolute error chart and the fact that the maximum absolute error was found at a short distance. In this case, the mean relative error (5.7%) was much higher than the rest of the collected values. In fact, the next distance tested (1.5 m) gave as a result a much more reduced error rate (2.4%), and from 2.5 m to 5 m, a distance interval in which the sensor showed the minimum absolute errors (Figure 9a), the relative results kept below 0.5% in every case (Figure 8b). Taking this result into account, this sensor would need at least 2.5 m to be precise, which could potentially limit its use in canopy characterization operations with narrow space in between the crop lines and wide trees, as the case of citrus, in which row spacing is typically set to 5 m [55]. Nevertheless, considering the absolute error found in the 2.5 m distance (<60 mm), there is no important effect in any operation carried out with agricultural machinery, such as pruning or pesticide application. In this last case, for example, the sensor measurements are used to define any canopy parameter that allows for the optimal spray dose calculation, which is generally known as “crop-adapted spraying” or “crop-adapted spray application” [56,57], based on the leaf amount and, therefore, adjusted to real requirements. For this purpose, potential mistakes at 60 mm for a tree crown volume calculation are significantly less important than the lack of vertical resolution caused by the sensor’s discrete measuring points, which divide the whole canopy into horizontal slices whose height corresponds to the spacing between sensors or measuring points [22]. In this sense, it could be better to mount a sensor of this type, in which 11 vertical measurement points can be obtained, rather than the typical configuration of a vertical pattern of ultrasonic or laser sensors [20,58].

Another important aspect is the data acquisition repeatability. Figure 8c shows the relationship between manually measured distances and sensor-measured ones. In the first place, the good correlation between both measurements is evident in the graph. If comparing the intersection points between manual and electronic measurements with the reference line in red which marks the perfect correlation between measurements (Figure 8c), the correlation is nearly perfect. The error bars in the graph indicate the 1000-fold standard error of the mean value for every pack of measurements, so that they can be seen properly. Therefore, it could be said that this variability is actually very small, even irrelevant. This circumstance can also be observed in the absolute error graph (Figure 8a), where the tiny error bars show the standard error for every sampled distance. This high repeatability is very desirable in any kind of sensor, not to mention one whose mission could be as relevant as planning the spray dose in commercial olive orchards.

#### 3.1.2. Sensor’s Cone Width Assessment

The sensor’s cone width for non-reflective and reflective targets are represented in Figure 9. As can be seen, both targets present very important cone width, especially with the highest sampling distance. Depending on the target, the cone radius ranged from 0.45 to 1.12 m in the case of the non-reflective target, and from 0.42 to 1.63 m in the case of the reflective one. If converting these values into diameter ones, they rise above 3 m in the case of the reflective target. It was also shown that the cone width did not present significant differences for the two directions considered (axis *x* and *y*, Figure 9). In fact, the small differences between the points obtained in both axes could be more due to the data acquisition methodology rather than any possible irregularity in the cone shape, as the curve shape is nearly identical in both targets.

If comparing both graphs (Figure 9a,b), it can be observed that the response was considerably different in both cases. Thus, while in the case of the reflective target (Figure 9b) there was a linear response, with maximum cone width of 3.26 m for 5 m distance, the case of the non-reflective target (Figure 9a) was much more irregular, but the cone with kept below 2.30 m for every sampled distance. The case of the reflective target presented two different slopes: from 0.5 to 2.0, there was a more abrupt increase, and from 2.0 to 4.5 m, there was a much smoother increase. Finally, for 5.0 m, the cone width increased markedly. In the case of the non-reflective target, the cone width grew abruptly from 0.5 to 2.0 m distance, and then kept relatively constant with non-important variations. There was a marked reduction in the cone width for the 4.5 m distance, which interrupted some sort of reduced increase trend from 2.5 m distance.

As mentioned before, the spot measurement is similar to that of the long-range ultrasonic sensor [22], but there is a very important advantage in this case: there is no interference, which makes it possible to have a higher vertical resolution.

#### 3.1.3. Sensor’s Accuracy with Density-Varying Target

**Figure 10:**
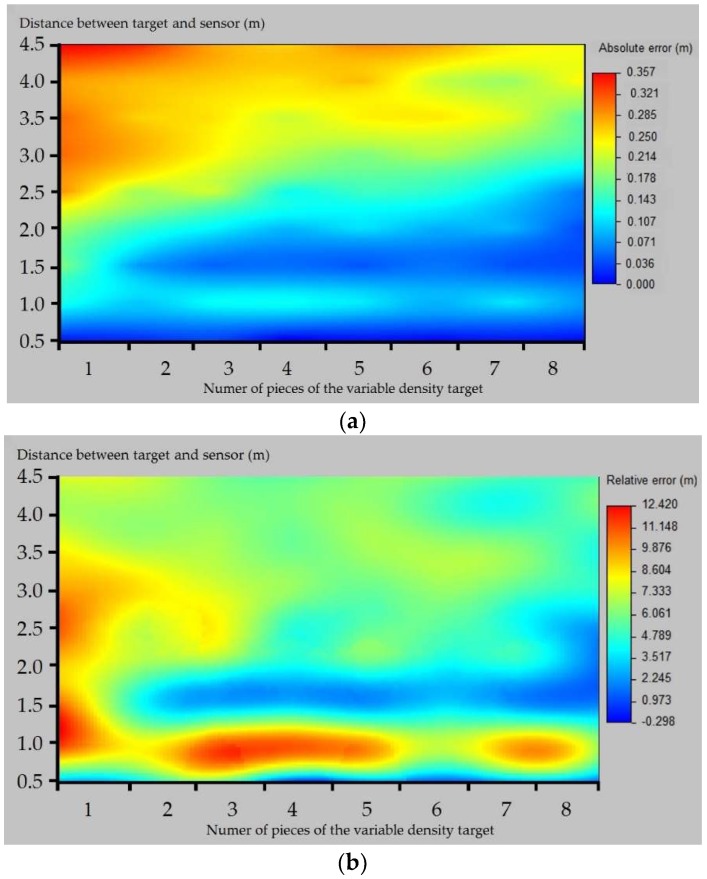
Average (**a**) absolute and (**b**) relative errors with density-varying target.

The absolute and relative errors produced by the sensor are included in Figure 10.

There is a strong influence of both factors included on the accuracy test. Thus, the higher the distance, the lower the accuracy, as shown in the decrease of the absolute and relative error in the lower part of the tables. As for the number of plastic sheets, the higher the number, the lower the error, which means that the target density affects the sensor’s accuracy. In general, the absolute error was well correlated with measurement distance, with gross errors at the highest distances that reached 0.36 m in the less favorable case (4.64 m distance and 1 plastic sheet, Figure 10a). Severe errors were found at the same distance and for long–medium distances for a low number of plastic pieces. By contrast, low errors were found at the shortest distance and for medium–short distances with a high number of sheets. Errors of 0.2–0.3 m could lead to under- or over-estimations in the canopy volume that could lead to mistakes on high-accuracy demanding operations, such as pesticide applications. Nevertheless, errors below 0.1 m are acceptable for this kind of operation, as there are other limitations related to the machinery employed that are more important in practice.

**Figure 11:**
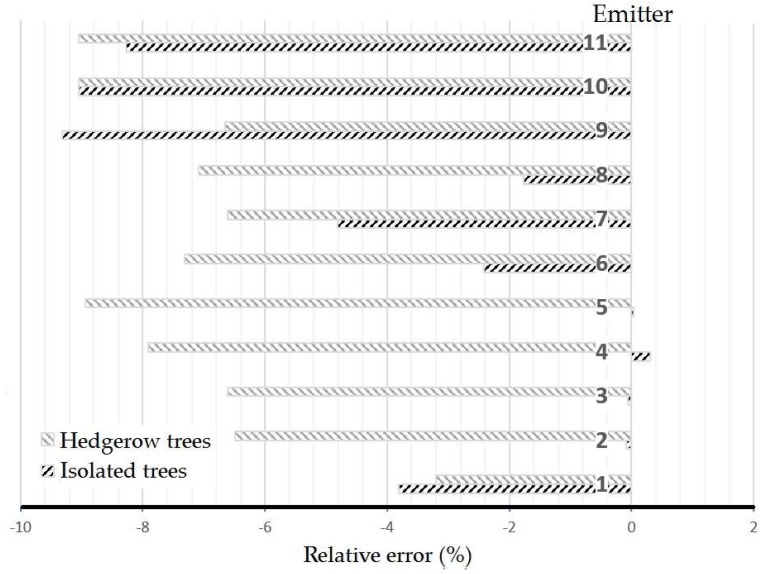
Mean values of the relative error of each emitters’ sensor.

If looking at the relative error, it generally remains under 10% in every case, except for the case of 1 plastic sheet in the lowest distances sampled and some isolated cases for the distance of 1.14 m, which could indicate some kind of malfunction in this measuring range, as the errors for the surrounding distances are much lower (Figure 10b). This fact indicates that the errors produced by the sensor are not very high in relation to the measured distance, which can be key when trying to characterize big-sized trees at long distances, as in the case of traditional olive orchards. Nevertheless, attention should be paid when pointing at low-leaf-density trees, as was shown in Figure 11b, where the lower the target density (correlated with the number of pieces), the poorer the sensor performance.

### 3.2. Field Results

The accuracy of each emitter for both cultivation systems is shown in Figure 11. As can be surmised from the figure, the errors are negative in nearly every case. This means, according to Equations (1) and (2), that the distances measured by the sensor were generally higher than those manually measured. This fact can be explained by the fact that the receivers need an amount of energy to read a certain distance, and the first leaves present a small surface for the light to bounce, meaning a small penetration of the emitted light into the canopy before being sent back to the receiver, which increases the measured distance in practice.

The mean relative errors (RE) found for the sensor were −3.09 ± 10.02% and −6.98 ± 9.76% in isolated and hedgerow trees, respectively. This error below 10% in general is comparable to those obtained by different authors with similar sensors in similar studies, even with LiDAR sensors [9]. In practice, it does not represent an important problem in the real field situations in olive orchards in which the resolution in any operation undertaken is generally low, so a very high characterization resolution is unneeded. The data acquisition time of the tested sensor (50 Hz) allows monitoring only 11 measurements very fast, and this may be used in several real-time field applications processed properly.

Analyzing emitter by emitter, the highest mean errors appeared in the highest positions, as emitters 9, 10 and 11 showed important mean errors, much above the results of the central ones. In addition, emitter 11 (Figure 3b) was oriented too vertically to reach the whole canopy of trees, so it gave measurements with value 65,535 indicating extreme difficult in identifying where some errors may be introduced.

On the other hand, emitters 2 to 5 presented errors below 1%, and in general emitters 1 to 8 gave errors below 5%. These emitters play a key role during canopy characterization because of their low and central position. A particular case was detected in emitters 2 to 5, which pointed to the trunk or low branches and generated errors in the extreme points. In general, it could be stated that emitters pointing to extreme low-density branches increased the measuring errors significantly. The hedgerow orchard, on the other hand, showed higher but more homogeneous mean errors (Figure 11).

Another important aspect of the measurements is their repeatability, given by the standard deviation of the mean value of every pack of measurements obtained by each emitter for every single sampling position. The whole pack of measurements taken by each emitter resulted in standard deviation values that are summarized in Table 1. As can be seen, the standard deviation values of the data obtained by every emitter are very similar, except for emitters 8 to 11, in which there is a considerable increase of about 40% with respect to the ones of emitters 1 to 7. The higher deviation concurs with the top zones of the trees where the canopy is less dense and may be due to the movement of the branches by external agents as wind. Nevertheless, the deviation is not important, remaining in general under 20 mm, which is fairly acceptable for the sensor’s intended application.

The evaluation carried out seems to indicate that the scanner could be used for multiple agricultural applications. Some useful applications could be the estimation of the canopy contour for pruning, automatic trunk detection for several applications, tree volume estimation for canopy spraying or tree contour delimitation for olive canopy shakers for harvesting. Nevertheless, this study only comprised the sensor’s accuracy assessment, and should be complemented with further specific studies regarding other different possible applications. Among them, its feasibility for canopy volume determination against high-accuracy methodologies such as LiDAR or digital photogrammetry, would be a high priority step for its utility. Therefore, our future studies will be oriented in this way.

## 4. Conclusions

The 2D laser scanner OMD8000-R2100-B16-2V15 was tested in laboratory and field trials, in real olive orchards, to check its accuracy and abilities to be used to determine canopy volumes in isolated and hedgerow trees. The following conclusions can be drawn:

- The sensor showed a high accuracy in the laboratory tests, with absolute errors under 60 mm and relative ones under 6%. This error increased at 1 m distance from the target, and rapidly decreased for further distances, which means that this sensor should work at, at least, 1.5 m away from the canopy. In general, from this distance up to 5 m, absolute errors decrease below 20 mm and relative errors below 1%. The sensor measurements are highly repetitive, with very low deviation in both laboratory and field conditions. This means that, in the same conditions, the sensor will give the same measurement with very low variation, which makes it reliable when aiming irregular targets, such as real canopies.
- The sensor’s cone width was considerable, reaching 3.2 m at 5 m distance. This could provide measurement mistakes when aiming at precise points, but loses importance because of the superposition of different emitters, which scan the canopy and, crucially, solve this inconvenience. Another important drawback, the overlap between consecutive emitters, does not take place because of the sensor’s construction. The reflectivity of the target crucially influences the sensor’s cone width, being much higher in the reflective ones, especially for long distances.
- The target density crucially affects the sensor’s accuracy, as with the measurement distance. Thus, the higher the density, the higher the accuracy. Exactly the opposite behavior was found for the distance, whose increase results in accuracy decrease. The aforementioned parameters did not offer a completely linear reduction of the accuracy, as the sensor presented some particularities in specific points, especially at 1 m sampling distance, where the relative errors rose importantly.
- The field trials showed the sensor’s accuracy when aiming at real olive canopies. The different emitters behaved differently according to the tree type: the isolated trees gave lower errors but higher heterogeneity, and the opposite in the hedgerow trees. In general, errors were below 10% and, in the case of the central and lower emitters in the isolated trees, they were nearly 0. The highest and lowest emitters presented problems for not measuring normally, as the first did not find a valid target and the second found many low-density branches that increased the error. The volume estimation methodologies resulted in different degrees of accuracy in both cases. While the intensive trees gave good results in terms of volume estimation by the studied sensor, the hedgerow tree volumes were underestimated. This could be solved by adjusting models against precise LiDAR sensors in future studies. The geometric estimations resulted in a severe over-estimation of the isolated tree volume and in an accurate estimation of the hedgerow tree volume. This could have an important limitation for irregular canopy profiles.
- The evaluated sensor offered a reasonable degree of accuracy for field operations, for example pesticide dose adjustment, pruning, or canopy contact harvesters. For volume estimation purposes, LiDAR sensors are more appropriate for their lower measurement errors. The multi-beam scanner sensor seems a valid alternative to manual methodologies or the ultrasonic sensors present in commercial sprayers, and allows for a proper profile definition for real-time use, not only in olives trees but also in other crops because of its operation principles.

## Figures and Tables

**Table 1:** Emitters mean standard deviation values.

Emitter	Standard Deviation (mm)
1	11.41
2	12.13
3	12.17
4	10.62
5	9.32
6	8.87
7	11.62
8	14.37
9	17.82
10	24.36
11	18.93
